# Ca^2+^/Calmodulin-Dependent Protein Kinase II (CaMKII) β-Dependent Phosphorylation of GABA_B1_ Triggers Lysosomal Degradation of GABA_B_ Receptors via Mind Bomb-2 (MIB2)-Mediated Lys-63-Linked Ubiquitination

**DOI:** 10.1007/s12035-018-1142-5

**Published:** 2018-06-07

**Authors:** Khaled Zemoura, Karthik Balakrishnan, Thomas Grampp, Dietmar Benke

**Affiliations:** 1 Institute of Pharmacology and Toxicology, University of Zurich, Winterthurerstrasse 190, 8057 Zurich, Switzerland; 2 Present Address: Picower Institute for Learning and Memory, Massachusetts Institute of Technology, 77 Massachusetts Avenue, Cambridge, MA 02139-4307 USA; 3 Neuroscience Center Zurich, University of Zurich and ETH Zurich, Winterthurerstrasse 190, CH-8057 Zurich, Switzerland; 4 Drug Discovery Network Zurich (DDNZ), Winterthurerstrasse 190, CH-8057 Zurich, Switzerland

**Keywords:** GABA receptor, CaMKII, Lysosome, Protein degradation, Ubiquitination, E3 ubiquitin ligase

## Abstract

The G protein-coupled GABA_B_ receptors, constituted from GABA_B1_ and GABA_B2_ subunits, are important regulators of neuronal excitability by mediating long-lasting inhibition. One factor that determines receptor availability and thereby the strength of inhibition is regulated protein degradation. GABA_B_ receptors are constitutively internalized from the plasma membrane and are either recycled to the cell surface or degraded in lysosomes*.* Lys-63-linked ubiquitination mediated by the E3 ligase Mind bomb-2 (MIB2) is the signal that sorts GABA_B_ receptors to lysosomes. However, it is unknown how Lys-63-linked ubiquitination and thereby lysosomal degradation of the receptors is regulated. Here, we show that Ca^2+^/calmodulin-dependent protein kinase II (CaMKII) promotes MIB2-mediated Lys-63-linked ubiquitination of GABA_B_ receptors*.* We found that inhibition of CaMKII in cultured rat cortical neurons increased cell surface GABA_B_ receptors, whereas overexpression of CaMKIIβ, but not CaMKIIα, decreased receptor levels. This effect was conveyed by Lys-63-linked ubiquitination of GABA_B1_ at multiple sites mediated by the E3 ligase MIB2. Inactivation of the CaMKII phosphorylation site on GABA_B1_(Ser-867) strongly reduced Lys-63-linked ubiquitination of GABA_B_ receptors and increased their cell surface expression, whereas the phosphomimetic mutant GABA_B1_(S867D) exhibited strongly increased Lys-63-linked ubiquitination and reduced cell surface expression. Finally, triggering lysosomal degradation of GABA_B_ receptors by sustained activation of glutamate receptors, a condition occurring in brain ischemia, was accompanied with a massive increase of GABA_B1_(Ser-867) phosphorylation-dependent Lys-63-linked ubiquitination of GABA_B_ receptors. These findings indicate that CaMKIIβ-dependent Lys-63-linked ubiquitination of GABA_B1_ at multiple sites controls sorting of GABA_B_ receptors to lysosomes for degradation under physiological and pathological condition.

## Introduction

γ-Aminobutyric acid (GABA), the main inhibitory neurotransmitter in the brain, activates the heterodimeric G protein-coupled GABA_B_ receptors, which are assembled from GABA_B1_ and GABA_B2_ subunits. GABA_B_ receptors are abundantly expressed in pre- and postsynaptic compartments of inhibitory as well as excitatory neurons to regulate their excitability [1]. GABA_B_ receptors are expressed by the majority of neurons and are involved in the regulation of virtually all important brain functions, such as neuronal network activity, synaptic plasticity, and neuronal development [2–5]. Accordingly, dysfunction of GABA_B_ receptor signaling has been implicated in a variety of neurological disorders [6–9].

For understanding the contribution of GABA_B_ receptors to physiological and pathological mechanisms, the elucidation of its regulation is essential. A main factor regulating GABA_B_ receptor signaling is the dynamic control of their cell surface expression. In this respect, protein degradation is one important mechanism that regulates receptor availability. The two main cellular protein degradation systems, which are proteasomes and lysosomes, contribute to the regulation of cell surface GABA_B_ receptors in different cellular compartments. In the endoplasmic reticulum (ER), the amount of newly synthesized GABA_B_ receptors that are trafficked to the cell surface is determined by proteasomal degradation via the ER-associated degradation (ERAD) machinery [10]. The activity state of the neuron controls the rate of Lys-48-linked ubiquitination of the GABA_B2_ subunit required for proteasomal receptor degradation [11]*.* In contrast, GABA_B_ receptors internalized from the cell surface are degraded in lysosomes [12–16]. Sorting the receptors to lysosomes is mediated most likely via the endosomal sorting complex required for transport (ESCRT) machinery [16], which targets ubiquitinated membrane proteins to lysosomes. Accordingly, lysosomal degradation of GABA_B_ receptors depends on Lys-63-linked ubiquitination of the GABA_B1_ subunit at multiple sites mediated by the E3 ubiquitin ligase MIB2 [17]. However, the mechanism that regulates Lys-63-linked ubiquitination of GABA_B1_, and thereby lysosomal degradation of the receptors, was unknown. Here, we show that phosphorylation of Ser-867 of GABA_B1_ by CaMKIIβ regulates the extent of MIB2-mediated K63-linked ubiquitination of GABA_B1_ and thereby the amount of lysosomal degradation.

## Materials and Methods

### Antibodies

Mouse anti-HA (clone HA-7, 1:1000 for immunofluorescence, 1:500 for in situ PLA, Sigma-Aldrich catalog no. H9658, lot no. 024M4773), rabbit CaMKII (1:500 for immunofluorescence, 1:100 for in situ PLA; Abcam catalog no. ab52476, lot no. GR181543-20), mouse EEA1 (1:100 for in situ PLA; BD Biosciences catalog no. 61047, lot no. 76250), rabbit GABA_B1b_ directed against the N-terminus of GABA_B1b_ (affinity-purified, 1:200 for immunofluorescence, custom made by GenScript) [18], rabbit GABA_B2_ directed against the N-terminus of GABA_B2_ (affinity-purified, 1:500 for immunofluorescence; custom made by GenScript) [19], guinea pig GABA_B2_ (1:500 for immunofluorescence; Millipore catalog no. AB2255, lot no. 2484228), mouse GABA_B1_ (1:50 for in situ PLA; NeuroMab, clone N93A/49, catalog no. 75-183), rabbit ubiquitin K48-specific (clone Apu2, 1:50 for in situ PLA; Millipore, catalog no. 05-1307, lot no. 2385989), rabbit ubiquitin K63-specific (clone Apu3, 1:50 for in situ PLA; Millipore, catalog no. 05-1308, lot no. 2575910), rabbit MIB2 (1: 1000 for immunofluorescence, 1:250 for in situ PLA; MyBioSource, catalog no. MBS2014413, lot no. A20160407515), mouse phosphoserine (PSR-45, 1:150 for in situ PLA; Sigma-Aldrich, catalog no. P5747, lot no. 014M4791V), goat Rab7 (1:250 for in situ PLA; Santa Cruz Biotechnology, catalog no. sc-11303, lot no. BO911), and mouse Rab11 (clone 47, 1:25 for in situ PLA; Millipore, catalog no. 05-853, lot no. JBC1868959). Secondary antibodies were labeled with either Alexa Fluor 488 (1:1000, Invitrogen), Cy-3 (1:500, Jackson ImmunoResearch Laboratories), or Cy-5 (1:300, Jackson ImmunoResearch Laboratories).

### Drugs

The following chemicals were used for this study: glutamate (50 μM, Sigma-Aldrich) and KN93 (10 μM, Tocris Bioscience). [^3^H]CGP 54626 (30 Ci/mmol) was purchased from ANAWA Trading SA and CGP 56999A was kindly provided by Novartis.

### Plasmids

The following plasmids were used for this study: HA-tagged GABA_B1a_ [20]; GABA_B2_ [21]; HA-tagged GABA_B1a_(K689/699R), GABA_B1a_(K893R), and GABA_B1a_(K961R) [17]; GFP-tagged CaMKIIα (Addgene plasmid 21226) [22] and its functionally inactive mutant GFP-tagged CaMKIIα(K42M) (here designated CaMKIIα(DN)) (kindly provided by U. Bayer, University of Colorado Denver-AMC); GFP-tagged CaMKIIβ (Addgene plasmid 21227) [22] and its functionally inactive mutant EGFP-tagged CaMKIIβ(K43R) (here designated CaMKIIβ(DN)) (Addgene plasmid 21225) [23]; HA-tagged ubiquitin (Addgene plasmid 17608), HA-tagged ubiquitin (KO) (Addgene plasmid 17603), HA-tagged ubiquitin (K63) (Addgene plasmid 17606), and HA-tagged ubiquitin (K48R) (Addgene plasmid 17604) [24]; and HA-tagged ubiquitin (K63R) (kindly provided by L.-Y. Liu-Chen, Temple University, Philadelphia, USA).

### Mutation of GABA_B1a_

Serine 867 in GABA_B1a_ was mutated either to alanine or to aspartate by GenScript.

### Culture and Transfection of Cortical Neurons

Primary cultures (co-cultures of neurons and glia cells) of the cerebral cortex were prepared from 18-day-old embryos of Wistar rats as described previously [25]. Neurons were used after 11 to 15 days in culture. Plasmid DNA was transfected into neurons by magnetofection using Lipofectamine 2000 (Invitrogen) and CombiMag (OZ Biosciences) exactly as specified in Buerli et al. [25]. In our hands, this method reliably yields 50–100 transfected neurons, when transfection was performed with 11–13-day-old cultures.

### Culture and Transfection of HEK 293 Cells

HEK (human embryonic kidney) 293 cells were cultured in Dulbecco’s modified Eagle’s medium (DMEM, Gibco Life Technologies) containing 10% fetal bovine serum (Gibco Life Technologies) and penicillin/streptomycin (Gibco Life Technologies). Plasmids were introduced into HEK 293 cells using the polyethylenimine method according to the jet-PEI protocol (Polyplus Transfection).

### Immunocytochemistry and Confocal Laser Scanning Microscopy

Immunofluorescence staining was done as described previously [13, 26]. For detection of total GABA_B_ receptors, neurons were fixed for 15–20 min at room temperature with 4% paraformaldehyde followed by permeabilization with 0.2% Triton X-100 and immunostaining. For analysis of cell surface GABA_B_ receptors, living neurons were incubated with antibodies recognizing the extracellularly located N-terminal domain of GABA_B1_ or GABA_B2_ for 1 h at 4 °C. After washing, the neurons were incubated with fluorophore-labeled secondary antibodies for 1 h at 4 °C and subsequently fixed with 4% paraformaldehyde.

Images of stained neurons were recorded by laser scanning confocal microscopy (LSM 510 Meta, LSM 700 or LSM 710; Zeiss). Five to eight optical sections spaced by 0.3 μm were taken with a ×40, ×63, or ×100 plan-fluar oil differential interference contrast objective (Zeiss) at a resolution of 1024 × 1024 pixels. Total and cell surface fluorescence signals were quantified using the ImageJ software as described previously [26]. To determine cell surface expression of GABA_B_ receptors, all optical sections were merged into one image. Then, the inner as well outer border of somatic staining was carefully outlined and the mean intensity values were measured. Values for cell surface signals were obtained by subtracting the values of the inner border from those of the outer border. For analysis of total GABA_B_ receptor expression, the somata of neurons were carefully outlined and the mean intensity values were measured. For each image, background staining was determined in an area containing no specific signal and subtracted from the mean intensity values. All values were normalized to the analyzed cell area.

Proteins overexpressed in neurons were either tagged with GFP or the HA epitope, which were used to identify transfected neurons and to judge the protein expression level. In case of overexpression studies, transfected neurons with comparable expression of the protein of interested were included into the analysis.

### In Situ Proximity Ligation Assay

In situ proximity ligation assay (PLA) is an antibody-based technology for the detection of protein-protein interactions and posttranslational modifications of proteins [27, 28]. We applied in situ PLA for the analysis of GABA_B_ receptor K48-linked or K63-linked ubiquitination, serine phosphorylation, and CaMKII/GABA_B_ receptor interaction as well as for the co-localization with the endosomal marker proteins EEA1, Rab7, and Rab11. In situ PLA was performed using Duolink PLA probes and detection reagents (Sigma-Aldrich) according to the manufacturer’s instructions as described previously [26]. Quantification was done by counting individual in situ PLA spots using the Image J software. The optical sections of each image stack were merged into one image and the number of maxima was determined after setting an appropriate noise tolerance (noise tolerance was kept constant for all images of an experiment). The number of spots was normalized to the area analyzed and to the expression level of GABA_B_ receptors. In some experiments, signals were too abundant to resolve individual spots precisely. In those cases, the fluorescence intensity of signals was determined.

### Internalization Assay

Neurons were placed on ice and cell surface GABA_B_ receptors were labeled with GABA_B2_ antibodies for 1 h at 4 °C. After extensive washes, neurons were incubated in 37 °C warm medium for exactly 1, 2, 3, 5, or 10 min to permit receptor endocytosis. Endocytosis was terminated by replacing the medium with ice-cold buffer. After incubation with secondary antibody for 1 h at 4 °C, the neurons were fixed with 4% PFA and remaining cell surface GABA_B_ receptors were determined by laser scanning confocal microscopy.

### Western Blot Analysis

For Western blot analysis, neuron/glia co-cultures were grown for 13–14 days on 6-cm culture dishes plated with 450,000 cells obtained from the cerebral cortex of E18 rat embryos. After incubation with KN93, cultures were immediately washed twice with ice-cold PBS, harvested, and homogenized by sonication. After protein determination using the Bradford protein assay (BioRad), the samples were incubated with Laemmli sample buffer (BioRad) for 2 h at 37 °C and aliquots containing 30 μg protein were subjected to sodium dodecyl sulfate-polyacrylamide gel electrophoresis (SDS-PAGE) using 7.5% mini-gels (Mini Protean 3, BioRad). Proteins were transferred onto nitrocellulose membranes in a Mini Trans-Blot Module (BioRad) at 365 mA for 120 min using 192 mM glycine, 25 mM Tris, 0.1% SDS, 20% methanol as transfer buffer. After blotting, the transferred proteins were stained with Amidoblack and immediately imaged using the E-box VX2 gel imager (Vilber). For immunodetection, the blots were blocked for 1–2 h in PBST (PBS pH 7.4, 0.05% Tween 20) containing 5% nonfat dry milk at room temperature, followed by incubation with antisera overnight at 4 °C in PBST containing 5% non-fat dry milk. The blots were then washed five times for 10 min with TBST and incubated with secondary antibodies conjugated to horseradish peroxidase for 1 h at room temperature. Following extensive washing (see above), immunoreactivity was detected by the chemoluminescence method (SuperSignal West Dura, Thermo Scientific) using a Fujifilm LAS-1000 imager. Immunoreactivity was quantified with the Image Studio software (LI-CORE Biosciences) and normalized to total protein in the corresponding lanes (determined by Amidoblack staining, see above).

### Radioligand Binding

For radioligand binding, 450,000 cells derived from the cerebral cortex of E18 rat embryos were plated onto 6-cm polylysine-coated culture dishes and kept in culture for 14 days. After drug incubation, cells were immediately washed two times with ice-cold PBS and harvested. Cells were resuspended in 500 μl binding buffer containing protease inhibitors (50 mM Tris pH 7.4, 2.5 mM CaCl_2_, complete mini, Sigma-Aldrich) and homogenized using a Potter S homogenizer (B. Braun Biotech International). Aliquots of the homogenate were incubated with 6.7 nM [^3^H]CGP 54626 in binding buffer for 90 min at room temperature. The incubation was terminated by rapid filtration onto glass fiber filters using a 12-channnel semi-automated cell harvester (Scatron) and washed with ice-cold binding buffer. Non-specific [^3^H]CGP 54626 binding was determined in the presence of 10 μM CGP 56999A. The radioactivity retained by the filters was determined by liquid scintillation counting using a Tricarb 2500 liquid scintillation analyzer.

### Statistics

The statistical analyses were done with GraphPad Prism 5. The tests used and *p* values are given in the figure legends. Differences were considered statistically significant when *p* < 0.05.

## Results

### CaMKIIβ Controls Expression of GABA_B_ Receptors

**Fig. 1:**
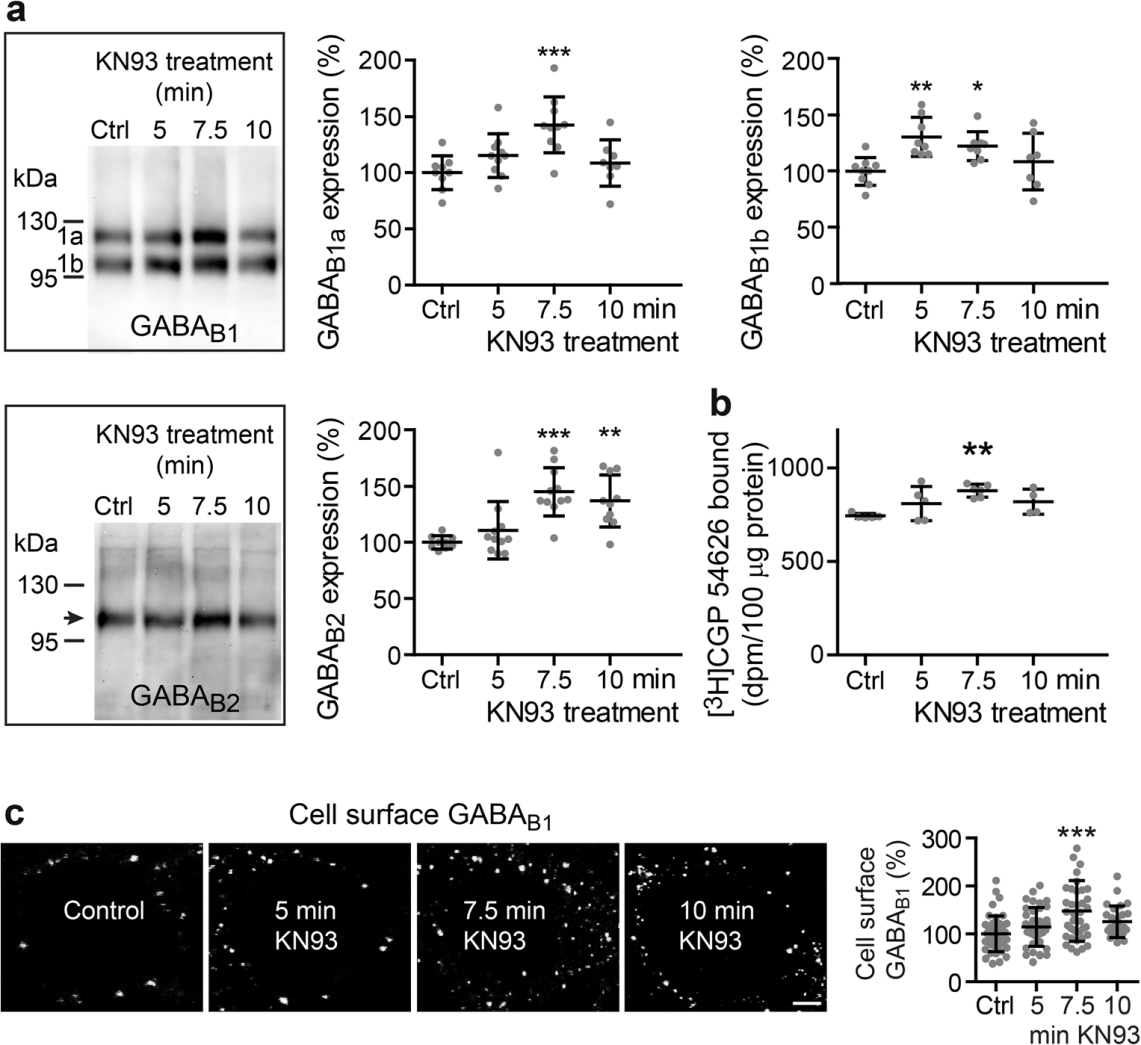
CaMKII regulates expression of GABA_B_ receptors. Neuronal cultures were incubated for the indicated times with the CaMKII inhibitor KN93 (10 μM) and tested for GABA_B_ receptor expression by Western blotting using antibodies against GABA_B1_ and GABA_B2_ (**a**) or radioligand binding with the GABA_B_ receptor antagonist [^3^H]CGP 54626 (**b**). In addition, cell surface expression of GABA_B_ receptors was analyzed by immunocytochemistry using GABA_B1_ antibodies (**c**). Untreated cultures served as controls. **a** Mean ± SD of 8–10 independent cultures for GABA_B1_ and 7–9 cultures for GABA_B2_; **b** Mean ± SD of 4–6 independent cultures; **c** Mean ± SD of 39–43 neurons from three independent experiments. Scale bar in representative immunofluorescence images depicted in **c** represents 5 μm. *p < 0.05, **p < 0.01, ***p < 0.001; one-way ANOVA, Dunnett’s multiple comparison test

Sustained activation of glutamate receptors, which occurs in cerebral ischemia, downregulates GABA_B_ receptors by preferentially sorting constitutively internalized receptors to lysosomes for degradation instead of recycling them to the cell surface [29–32]. Phosphorylation of GABA_B1_ at Ser-867 by CaMKII plays a key role in this mechanism [29]. However, it was unknown whether CaMKII is also involved in the regulation of GABA_B_ receptor expression under normal physiological conditions. Therefore, we tested whether inhibition of CaMKII by KN93 affects total expression of GABA_B_ receptors in cultured cortical neurons. Incubation of neurons with KN93 transiently increased the expression of GABA_B1_ and GABA_B2_, which peaked at about 7.5 min as tested by Western blotting (Fig. 1a). This was confirmed by radioligand binding experiments on homogenates prepared from the cultures using the GABA_B_ receptor antagonist [^3^H]CGP 54626 (Fig. 1b). Finally, we monitored cell surface expression of GABA_B_ receptors by staining living neurons at 4 °C with antibodies directed against the extracellular located N-terminal domain of the receptors. In line with the experiments on total receptor expression, incubation of neurons with KN93 for 7.5 min considerably increased the level of GABA_B_ receptors at the cell surface (148 ± 64% of control; Fig. 1c). The cause for the transient nature of blocking CaMKII on cell surface expression of the receptors is currently unclear. It might well be that a so far unknown homeostatic response of the neurons downregulates the increased cell surface receptors to normal levels. However, our results clearly indicate that the expression of the receptors in the plasma membrane is indeed controlled by CaMKII under basal conditions.

**Fig. 2:**
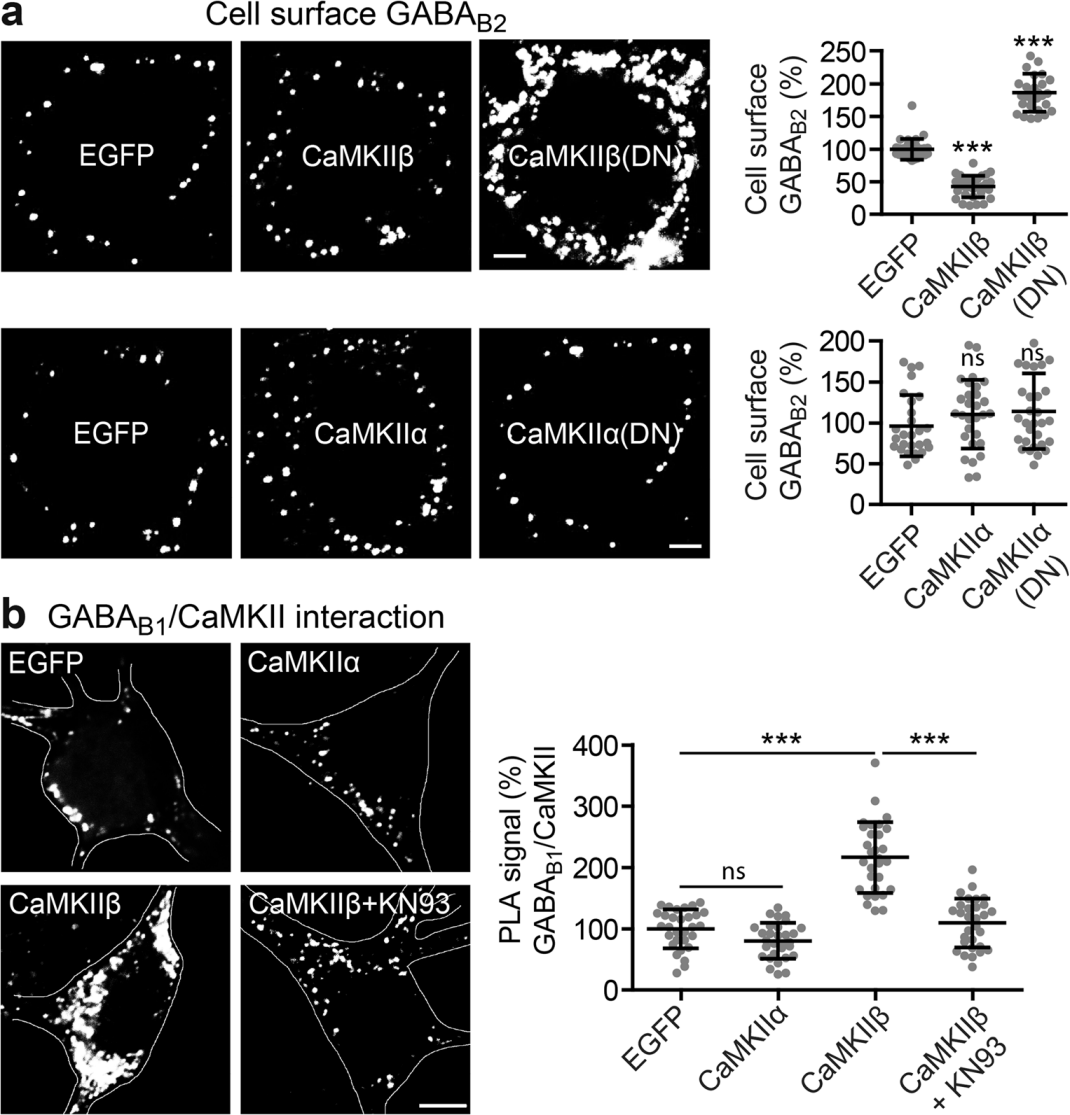
CaMKIIβ interacts in an activity-dependent manner with GABA_B_ receptors to regulate their cell surface expression. **a** CaMKIIβ but not CaMKIIα regulates cell surface expression of GABA_B_ receptors. Cortical neurons were transfected with either EGFP (control), CaMKIIα, CaMKIIβ, or their functionally inactive mutants CaMKIIβ(DN) or CaMKIIα(DN). Two days after transfection, cell surface expression of GABA_B_ receptors was assessed using GABA_B2_ antibodies. Transfected neurons were identified via the GFP-tag of transfected CaMKII constructs (not shown). Left, representative images of the soma of stained neurons (scale bar, 5 μm). Right, the graphs show the quantification of fluorescence intensities. Fluorescence intensities for GABA_B2_ in EGFP-transfected neurons were set to 100%. The data represent the mean ± SD of 26–30 neurons from three independent experiments. ***p < 0.001, ^ns^*p* > 0.05; one-way ANOVA, Dunnett’s multiple comparison test. **b** CaMKIIβ interacts with GABA_B_ receptors in an activity-dependent manner. Neurons were transfected with either EGFP (control), CaMKIIα, or CaMKIIβ and tested for interaction of CaMKII with GABA_B_ receptors in the presence of absence of KN93 by in situ PLA using antibodies directed against CaMKII and GABA_B1_. Left, representative images of in situ PLA signals (white dots) in the soma of neurons (scale bar, 5 μm). Right, the graph shows the quantification PLA signals. The data represent the mean ± SD of 30 neurons per condition from three independent experiments. ***p < 0.001, ^ns^*p* > 0.05; one-way ANOVA, Dunnett’s multiple comparison test

CaMKIIα and CaMKIIβ are the main CaMKII isoforms expressed in neurons [33]. We therefore tested which isoform is involved in the regulation of GABA_B_ receptors by transfecting neurons with either CaMKIIα, CaMKIIβ, or their functionally inactive mutants (CaMKIIα(DN), CaMKIIβ(DN)) and determined cell surface expression of GABA_B_ receptors using GABA_B2_ antibodies. While transfecting CaMKIIα or CaMKIIα(DN) did not affect cell surface expression of GABA_B_ receptors, overexpression of CaMKIIβ decreased (43 ± 17% of EGFP-transfected control neurons, Fig. 2a) and its nonfunctional mutant CaMKIIβ(DN) increased cell surface levels of receptors (186 ± 29% of EGFP-transfected control neurons, Fig. 2b).

It was previously shown that CaMKII interacts with GABA_B1_ [29]. Next, we tested whether CaMKIIβ or CaMKIIα interacts with GABA_B_ receptors under basal conditions by overexpressing either CaMKIIα or CaMKIIβ in neurons and testing for changes in interaction levels with GABA_B_ receptors using in situ PLA with antibodies against GABA_B1_ and CaMKII (Fig. 2b). In line with our experiments described above, overexpression of CaMKIIα did not affect the level of GABA_B_ receptor/CaMKII interaction (PLA signal EGFP-transfected control, 100 ± 32%; CaMKIIα, 80 ± 30%), but transfection of CaMKIIβ significantly increased the extent of interaction (216 ± 58% of control). The interaction of CaMKIIβ with GABA_B_ receptors was activity-dependent as blocking CaMKII with KN93 reduced the interaction level in neurons transfected with CaMKIIβ (PLA signal. 110 ± 40% of control).

These results indicate that CaMKIIβ interacts with GABA_B_ receptors in an activity-dependent manner to regulate the cell surface expression of the receptors.

### CaMKII Triggers Sorting of GABA_B_ Receptors to Lysosomal Degradation

**Fig. 3:**
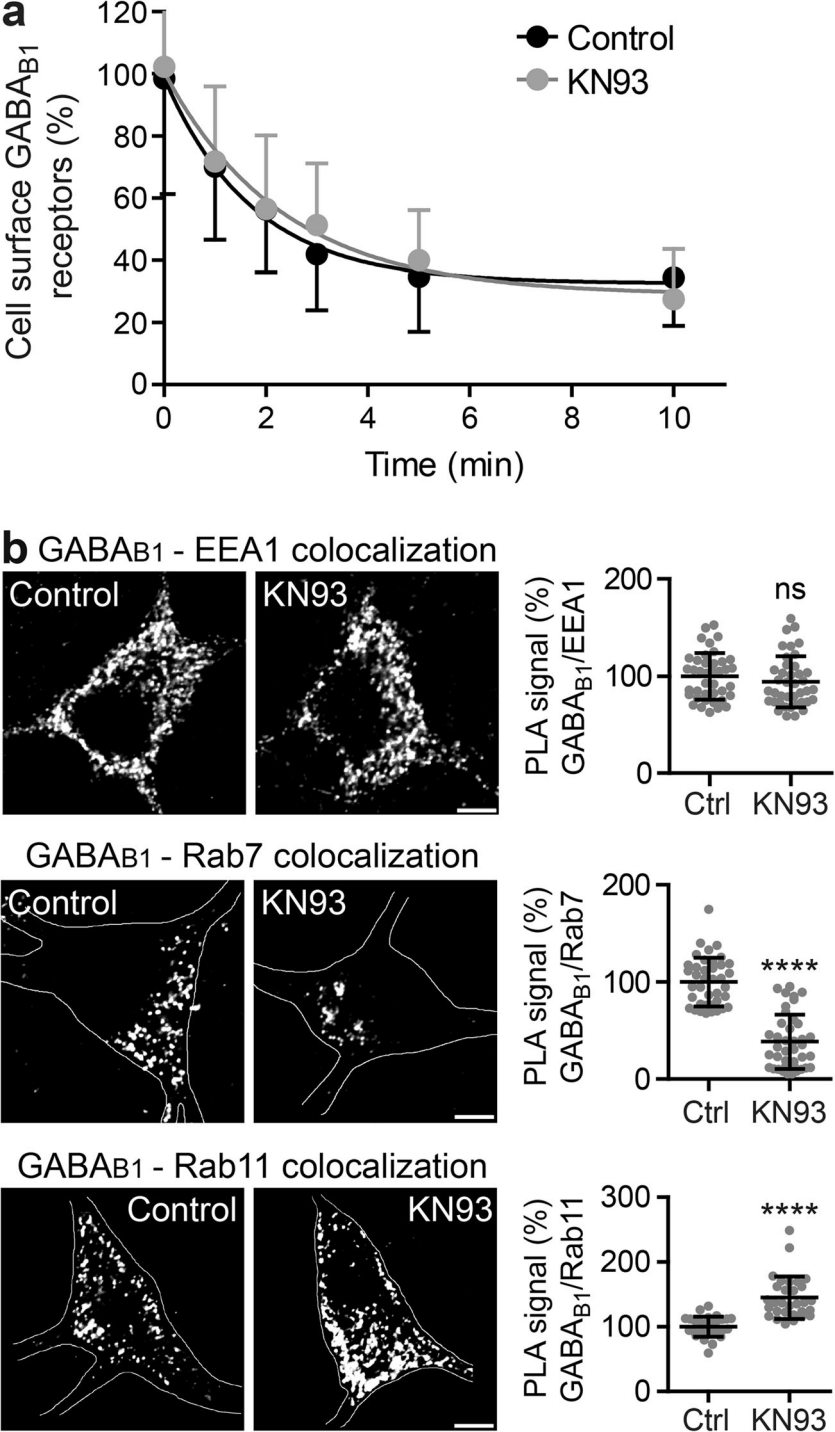
CaMKII triggers sorting of GABA_B_ receptors to lysosomes. **a** Blocking CaMKII activity does not affect internalization of GABA_B_ receptors. GABA_B2_ antibody-labeled cell surface receptors of living neurons were incubated for the indicated time intervals at 37 °C to permit endocytosis. Subsequently, remaining cell surface GABA_B2_-labeled receptors were quantified by immunofluorescence microscopy. The immunofluorescence signals of neurons at time 0 (not exposed to 37 °C) were set to 100%. Data were fitted by nonlinear regression to one phase decay. The data represent the mean ± SD of 35–46 neurons per time point from two independent experiments. **b** Blocking CaMKII activity decreased the co-localization of GABA_B_ receptors with Rab7 and increased the co-localization with Rab11. Neurons were either treated or not with KN93 and subjected to in situ PLA using antibodies directed against GABA_B1_ and Rab11 (marker for recycling endosomes), Rab7 (marker for the lysosomal pathway), or EEA1 (marker for early endosomes), respectively. Left, representative images (scale bar 5 μm). Right, quantification of PLA signals (white dots in images). The PLA signals were normalized to the cell area. The control condition was set to 100%. The data represent the mean ± SD of 30 neurons (Rab11) and 40 neurons (Rab7, EEA1) from two independent experiments. ****p < 0.0001, ^ns^*p* > 0.05; two-tailed unpaired *t* test

It had been suggested that phosphorylation of GABA_B1_ Ser-867 by CaMKII may mediate internalization of the receptors [29]. We therefore tested whether inhibition of CaMKII by KN93 affects the internalization of GABA_B_ receptors. However, blocking CaMKII with KN93 did affect neither the kinetics (half live; control, 1.2 min; KN93, 1.6 min) nor the extent (control, 64 ± 19%; KN93, 65 ± 20%) of receptor internalization (Fig. 3a).

To gain insight into the mechanism that is affected by CaMKII inhibition, we tested the co-localization of GABA_B_ receptors with the endosomal marker proteins EEA1 (marker for early endosomes), Rab7 (marker for late endosomes and lysosomes), and Rab11 (marker for recycling endosomes) [34] using in situ PLA. Inhibition of CaMKII activity in neurons with KN93 did not affect the co-localization of GABA_B_ receptors with EEA1 (94 ± 26% of control; Fig. 3b), supporting our observation of an unchanged internalization rate of the receptors. Instead, blocking CaMKII decreased the co-localization of GABA_B_ receptors with Rab7 (39 ± 28% of control; Fig. 3b) and increased the co-localization of GABA_B_ receptors with Rab11 (145 ± 32% of control; Fig. 3b). Thus, inhibition of CaMKII appears to prevent recruitment of GABA_B_ receptors to the lysosomal degradation pathway (Rab7) and increase recycling of the receptors (Rab11). This observation indicates that basal CaMKII activity is involved in targeting GABA_B_ receptors to lysosomes for degradation.

### CaMKIIβ Promotes Lys-63-Linked Ubiquitination of GABA_B_ Receptors

The results so far support the hypothesis that CaMKIIβ regulates lysosomal degradation of GABA_B_ receptors. We had previously shown that lysosomal degradation of GABA_B_ receptors depends on Lys-63-linked ubiquitination of GABA_B1_ at multiple sites via the E3 ligase MIB2 [17]. It was therefore likely that CaMKIIβ controls Lys-63-linked ubiquitination of GABA_B_ receptors.

**Fig. 4:**
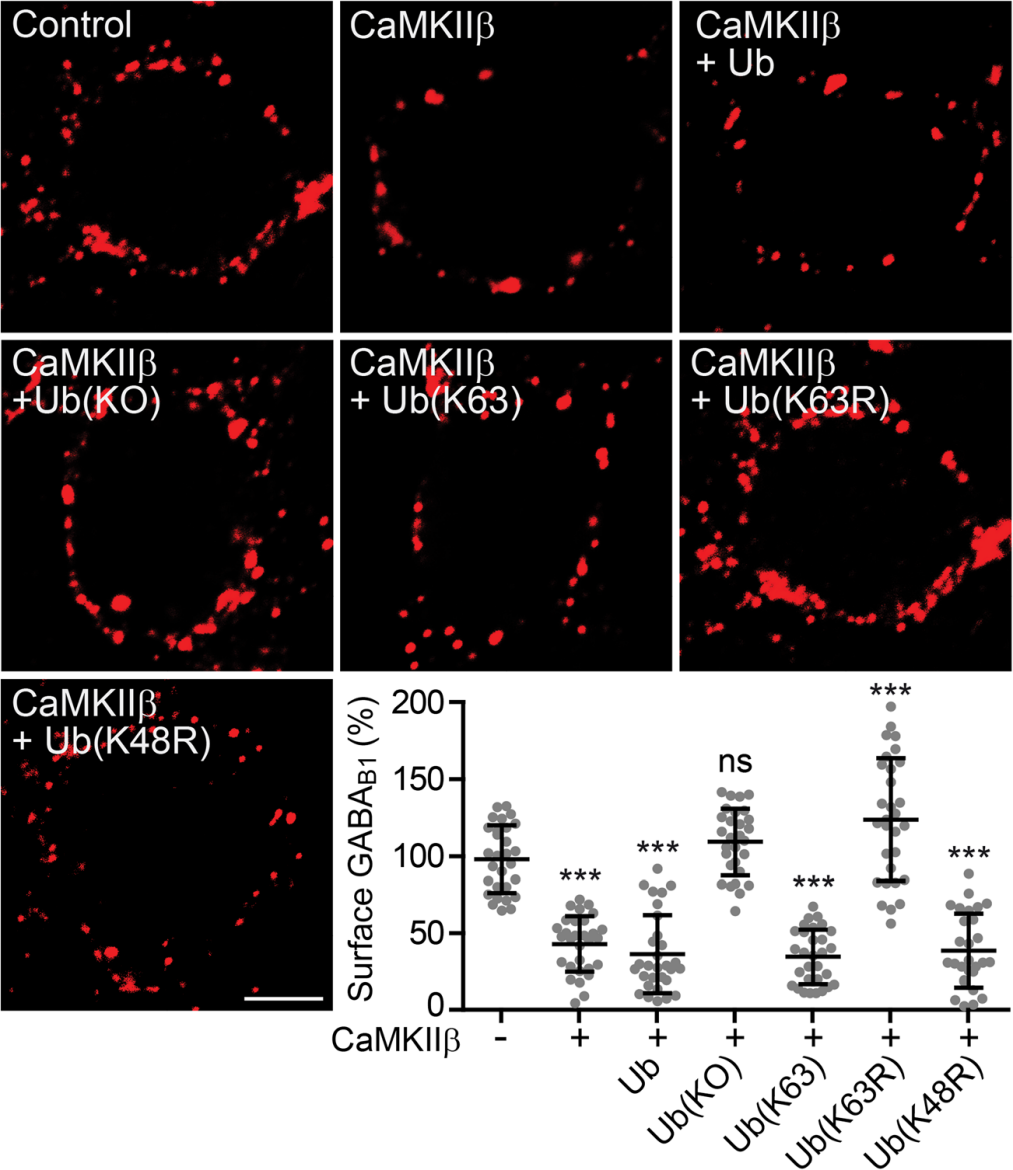
CaMKIIβ-induced downregulation of cell surface GABA_B_ receptors depends on Lys-63-linked ubiquitination. Neurons were transfected with either EGFP (control), CaMKIIβ, or CaMKIIβ plus ubiquitin (Ub) or one of its mutants (Ub(K63): only Lys-63 linkages, Ub(K63R): no Lys-63 linkages, Ub(K48R): no Lys-48 linkages, Ub(KO): no polyubiquitin linkages (only mono-ubiquitination). Two days after transfection, neurons were stained for GABA_B_ receptors using GABA_B2_ antibodies. Only interference with K63-linked ubiquitination prevented CaMKIIβ-mediated downregulation of the receptors. Representative images of stained neuronal somata (scale bar, 5 μm). The scatter plot shows quantification of fluorescence intensities. The fluorescence signal of neurons transfected with EGFP was set to 100%. The data represent the mean ± SD of 28–30 neurons from two independent experiments. ***p < 0.001, ^ns^*p* > 0.05; one-way ANOVA, Dunnett’s multiple comparison test

To test this hypothesis, we first analyzed the effect of various ubiquitin mutants on the downregulation of cell surface GABA_B_ receptors induced by overexpression of CaMKIIβ. Overexpression of CaMKIIβ in neurons reduced cell surface GABA_B_ receptors to 43 ± 18% of control neurons transfected with EGFP (Fig. 4). Additional overexpression of wild-type ubiquitin (Ub, 36 ± 25% of control) and a ubiquitin mutant that permits only Lys-63-linked ubiquitination (Ub(K63), 35 ± 18% of control) as well as a mutant that prevents Lys-48-linked ubiquitination (Ub(K48R), 39 ± 24% of control) did not affect CaMKIIβ-induced downregulation of the receptors (43 ± 18% of control). However, overexpressing a ubiquitin mutant in which all lysine residues were mutated to arginine to entirely block polyubiquitination (Ub(KO), 109 ± 22% of control) as well as a ubiquitin mutant that specifically prevents Lys-63-linked ubiquitination (Ub(K63R), 124 ± 40% of control) completely prevented downregulation of the receptors or even increased cell surface expression of the receptors (Fig. 4). These results indicate that downregulation of GABA_B_ receptors induced by overexpression of CaMKIIβ is mediated by Lys-63-linked ubiquitination.

**Fig. 5:**
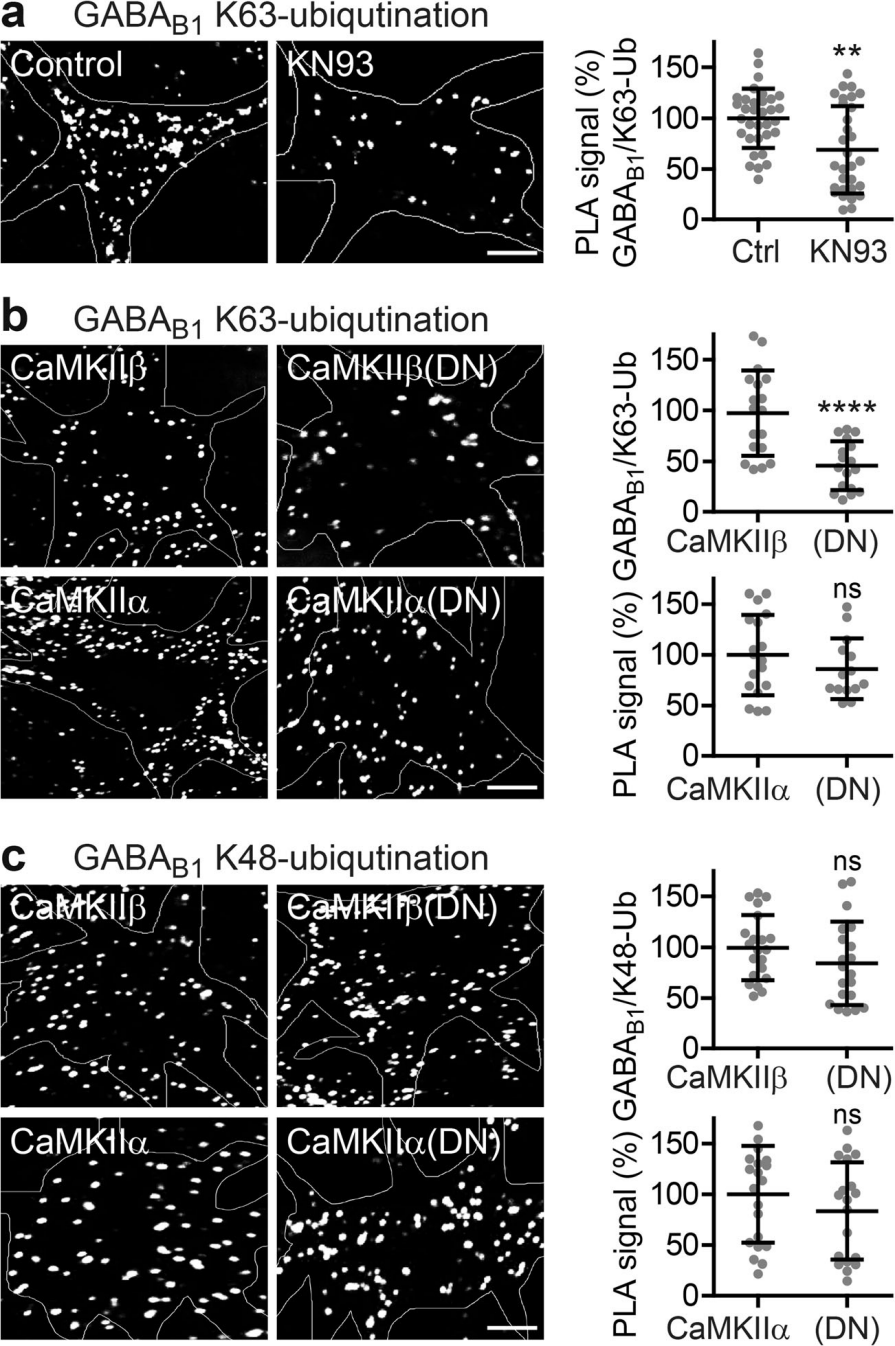
CaMKII regulates K63-linked ubiquitination of GABA_B_ receptors. **a** Inhibition of CaMKII activity reduced K63-linked ubiquitination of GABA_B_ receptors. Neurons were incubated for 7.5 min in the absence (control) or presence of KN93 and analyzed for K63-linked ubiquitination of GABA_B_ receptor by in situ PLA using antibodies directed against GABA_B1_- and K63-linked ubiquitin (white dots in representative images, scale bar 5 μm). Right, quantification of in situ PLA signals. The data represent the mean ± SD of 33 (control) and 30 (KN93) neurons from two independent experiments. **p < 0.01, two-tailed unpaired *t* test. **b**, **c** CaMKIIβ promotes Lys-63-linked ubiquitination of GABA_B_ receptors. Neurons were transfected with either CaMKIIα or CaMKIIβ or their functionally inactive mutants CaMKIIα(DN) or CaMKIIβ(DN). After 2 days, GABA_B_ receptors were tested for Lys-63-linked (**b**) or Lys-48-linked (**c**) ubiquitination by in situ PLA using antibodies directed against GABA_B1_ and Lys-63 or Lys-48 ubiquitin. Left, representative images depicting PLA signals (white dots, scale bars 5 μm). Right, quantification of PLA signals. The data represent the mean ± SD of 17–21 neurons from two independent experiments. **p < 0.01, ****p < 0.0001, ^ns^*p* > 0.05; two-tailed unpaired *t* test

Next, we tested for direct CaMKIIβ-induced Lys-63-linked ubiquitination of GABA_B_ receptors using in situ PLA and antibodies directed against GABA_B1_ and Lys-63-linked ubiquitin. Inhibition of CaMKII with KN93 significantly reduced basal Lys-63-linked ubiquitination of the receptor (69 ± 43% of control, Fig. 5a). In addition, overexpression of the functionally inactive CaMKIIβ mutant CaMKIIβ(DN) in neurons considerably reduced Lys-63-linked ubiquitination of the receptors (46 ± 24%, Fig. 5b) as compared to neurons transfected with wild-type CaMKIIβ. In contrast, overexpression of the functionally inactive mutant of CaMKIIα (CaMKIIα(DΝ)) had no effect on the level of Lys-63-linked ubiquitinated GABA_B_ receptors as compared to wild-type CaMKIIα. Finally, overexpression of neither CaMKIIα(DN) nor CaMKIIβ(DN) affected Lys-48-linked ubiquitination of the receptors, which is required for their proteasomal degradation (Fig. 5c). These results indicate that CaMKIIβ is involved in the regulation of Lys-63-linked ubiquitination of GABA_B_ receptors.

**Fig. 6:**
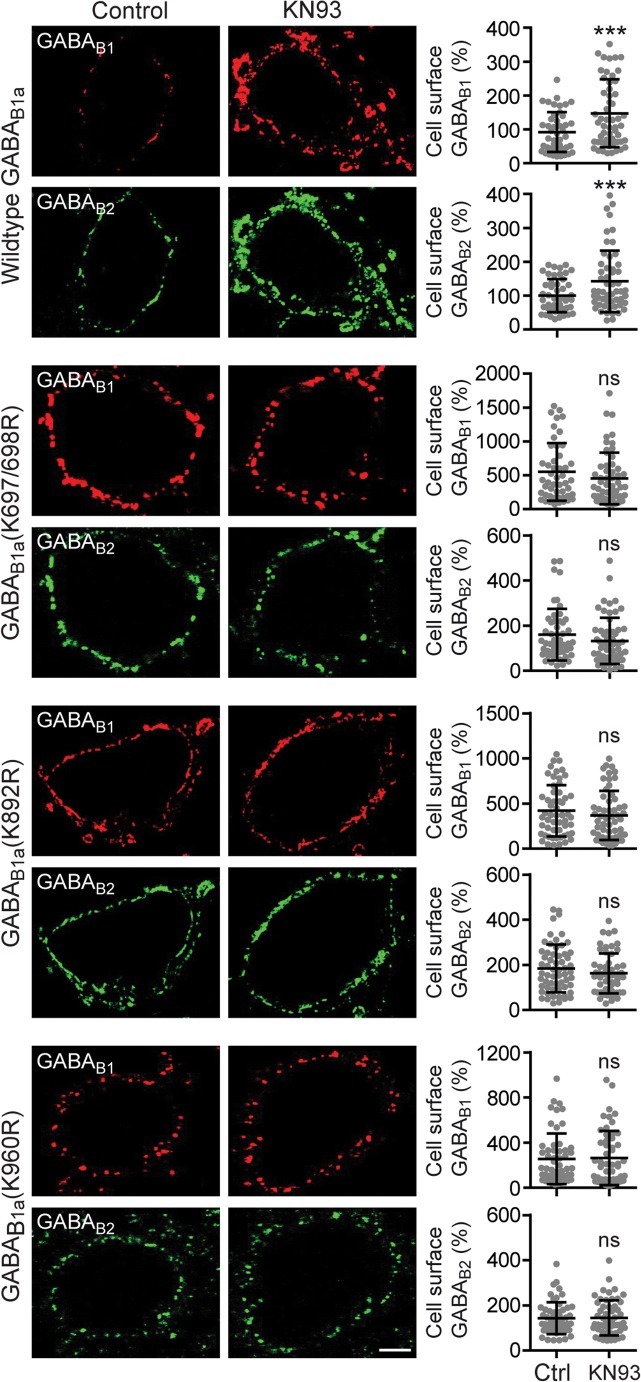
Blocking CaMKII did not affect the expression levels of GABA_B1a_(K->R) mutants. Neurons were transfected with HA-tagged wild-type GABA_B1a_ or HA-tagged GABA_B1a_ (K->R) mutants along with GABA_B2_ and tested for cell surface expression of GABA_B1_ and GABA_B2_ after treating the neurons with 10 μM KN93 for 7.5 min. Left, representative images of untreated neurons (control, left panels) and of neurons treated with KN93 (right panels, scale bar 5 μm). The corresponding graphs show the quantification of fluorescence signals. The fluorescence intensity of neurons transfected with wild-type GABA_B1_ from untreated neurons (control) was set to 100%. The data represent the mean ± SD of 45–60 neurons per experimental condition derived from three independent experiments. Please note that the considerably lower increase in GABA_B2_ cell surface expression as compared with mutant GABA_B1_ was due to the fact that in the case of GABA_B1_, only transfected subunits were detected (HA-tagged) but in the case of GABA_B2_, transfected as well as endogenously expressed subunits were detected. ***p < 0.001, ^ns^*p* > 0.05; two-tailed unpaired *t* test

We recently identified in GABA_B1_ several lysine residues (Lys-697/698, Lys-892, and Lys-960) that are substrates for Lys-63-linked ubiquitination and important for targeting the receptors to lysosomal degradation [17]. To further substantiate the finding that CaMKII regulates lysosomal degradation of GABA_B_ receptors by promoting Lys-63-linked ubiquitination of GABA_B1_, we tested whether blocking CaMKII affects cell surface expression of three GABA_B1a_(K->R) mutants in which Lys-697/698, Lys-892, or Lys-960 was mutated to arginine (GABA_B1a_(K697/698R), GABA_B1a_(K892R), and GABA_B1a_(K960R)). In contrast to receptors containing wild-type GABA_B1_, which displayed increased cell surface expression upon treatment with KN93 (GABA_B1_: 164 ± 149%, GABA_B2_: 142 ± 91% of control; Fig. 6), the expression level of receptors containing any of the three GABA_B1a_(K->R) mutants remained unaffected by inhibition of CaMKII (GABA_B1a_(K697/698R), control: 552 ± 426%, KN93: 490 ± 456%; GABA_B1a_(K892R), control: 421 ± 284%, KN93: 370 ± 272%; GABA_B1a_ (K960R), control: 273 ± 256%, KN93: 281 ± 270% of wild-type control; GABA_B2_ co-expressed with GABA_B1a_ (K697/698R), control: 160 ± 115%, KN93: 140 ± 117%; GABA_B2_ co-expressed with GABA_B1a_(K892R), control: 184 ± 106%, KN93: 163 ± 88%; GABA_B2_ co-expressed with GABA_B1a_(K960R), control: 147 ± 84%, KN93: 145 ± 78% of wild-type control; Fig. 6). In conclusion, these findings suggest that CaMKII is involved in K63-linked ubiquitination of GABA_B1_ at K697/698 and K892 as well as K960.

### CaMKII Regulates Cell Surface Receptors by Promoting MIB2-Mediated Lys-63-Linked Ubiquitination via Phosphorylation of GABA_B1_ at Ser-867

**Fig. 7:**
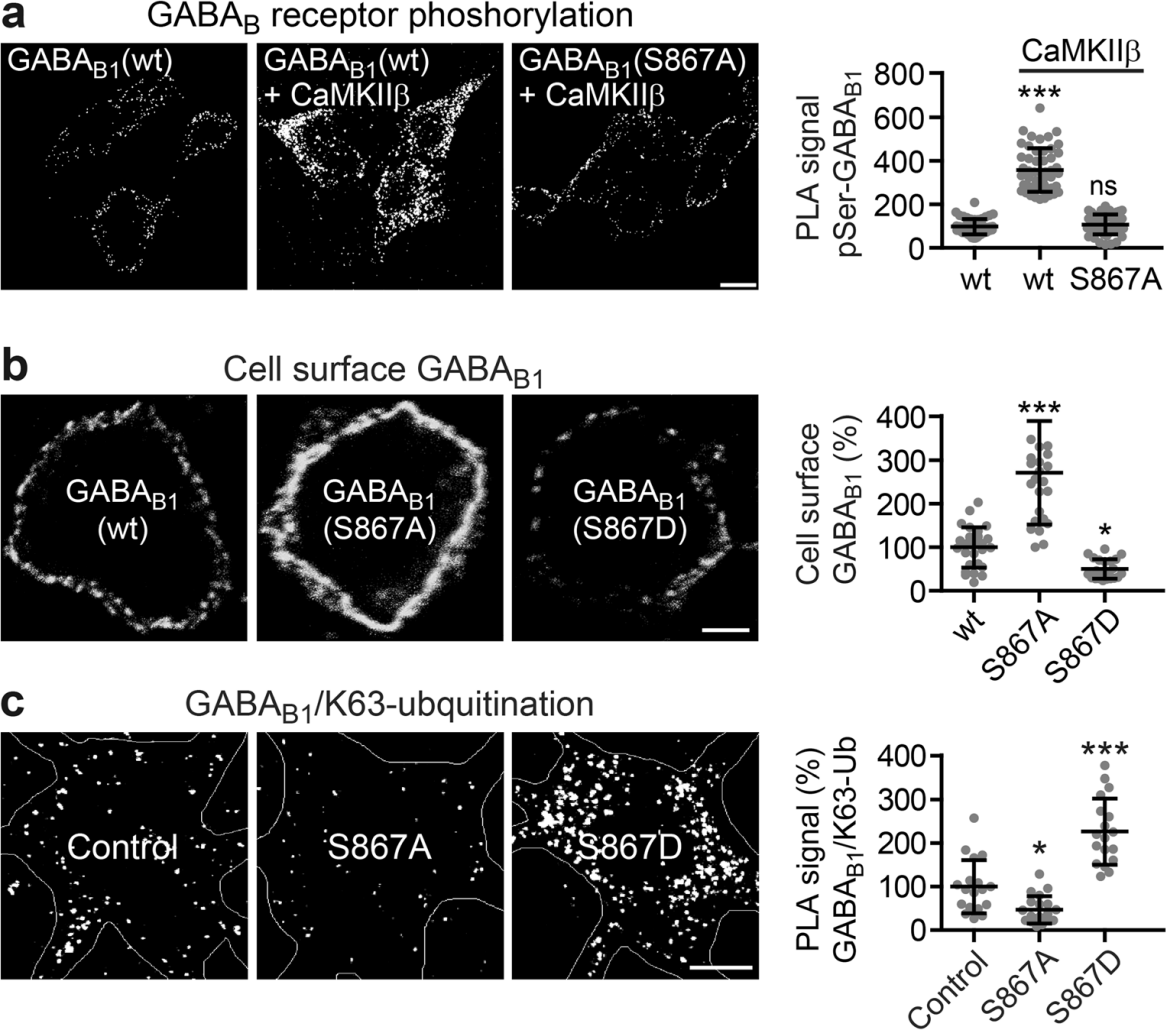
Phosphorylation of GABA_B1_ Ser-867 controls cell surface expression and Lys-63-linked ubiquitination of GABA_B_ receptors. **a** CaMKIIβ phosphorylates GABA_B1_ at Ser-867. HEK293 cells were transfected either with GABA_B1_/GABA_B2_ with or without CaMKIIβ or with GABA_B1_(S867A)/GABA_B2_ and CaMKIIβ. Two days after transfection, the cells were tested for serine phosphorylation by in situ PLA using antibodies directed against GABA_B1_ and phosphoserine. Left, representative images (scale bar, 10 μm; *wt*, wild type). Right, quantification of PLA signals (white dots in images). The PLA signals were normalized to the cell area as well as to the expression level of GABA_B1_. The data represent the mean ± SD of 50 cells from two independent experiments. ***p < 0.001, ^ns^*p* > 0.05; one-way ANOVA, Dunnett’s multiple comparison test. **b**, **c** Inactivation or constitutively mimicking phosphorylation of GABA_B1_ Ser-867 affects cell surface expression (**b**) as well as Lys-63-linked ubiquitination (**c**) of the receptors. Neurons were transfected with wild-type HA-tagged GABA_B1a_ (control), with the phosphorylation-deficient HA-tagged GABA_B1a_(S867A) mutant or with the phosphomimetic HA-tagged GABA_B1a_(S867D) mutant along with GABA_B2_ and tested for cell surface expression using HA antibodies (**b**) as well as for Lys63-linked ubiquitination by in situ PLA with HA and Lys-63 antibodies (**c**). Left, representative images (scale bars, 5 μm). Right, quantification of fluorescence intensities (**b**) and PLA signals (**c**). The data represent the mean ± SD of 23–27 (**b**) and 18–20 (**c**) neurons from two independent experiments. *p < 0.05, ***p < 0.001; one-way ANOVA, Dunnett’s multiple comparison test

CaMKII phosphorylates GABA_B1_ at Ser-867 [29]. To prove that direct phosphorylation of Ser-867 in GABA_B1_ is involved in the regulation of GABA_B_ receptor cell surface expression, we silenced this phosphorylation site by mutating it to alanine (GABA_B1_(S867A)) or rendered the site permanently active by mutating it to aspartate (GABA_B1_(S867D)). First, we verified that CaMKIIβ indeed phosphorylates GABA_B1_ at Ser-867 by in situ PLA using antibodies directed against GABA_B1_ and phosphoserine. Upon expression in HEK 293 cells, wild-type GABA_B_ receptors displayed a basal level of serine phosphorylation, which was more than threefold enhanced by co-expression with CaMKIIβ (PLA signal wt-GABA_B1_: 98 ± 5, GABA_B1_+CaMKIIβ: 358 ± 14; Fig. 7a). In contrast, GABA_B_ receptors in which Ser-867 of GABA_B1_ was inactivated by mutation to alanine, only basal level of serine phosphorylation was detected in the presence of CaMKIIβ (PLA signal GABA_B1_(S867A)+CaMKIIβ: 108 ± 6 compared to wt-GABA_B1_: 98 ± 5, Fig. 7a). These results confirm that CaMKIIβ specifically phosphorylates GABA_B1_ at Ser-867.

Next, we tested the effect of inactivating or permanently mimicking phosphorylation of GABA_B1_ Ser-867 on cell surface expression of the receptors by expressing GABA_B1_(S867A) or GABA_B1_(S867D) in cortical neurons. Inactivation of GABA_B1_ Ser-867 considerably increased cell surface expression of receptors containing GABA_B1_(S867A) (271 ± 119% of control, Fig. 7b) upon transfection in neurons, whereas receptors containing the phosphomimetic GABA_B1_(S867D) mutant were expressed at reduced levels (50 ± 22% of control, Fig. 7b). In line with this finding, receptors containing the CaMKII phosphorylation-deficient GABA_B1_(S867A) mutant exhibited reduced K63-linked ubiquitination (47 ± 32% of control, Fig. 7c), while receptors containing the phosphomimetic GABA_B1_(S867D) mutant showed a considerably higher level of Lys-63-linked ubiquitination (226 ± 76% of control, Fig. 7c). These findings suggest that CaMKII promotes Lys-63-linked ubiquitination of GABA_B_ receptors via phosphorylation of serine 867 in GABA_B1_.

**Fig. 8:**
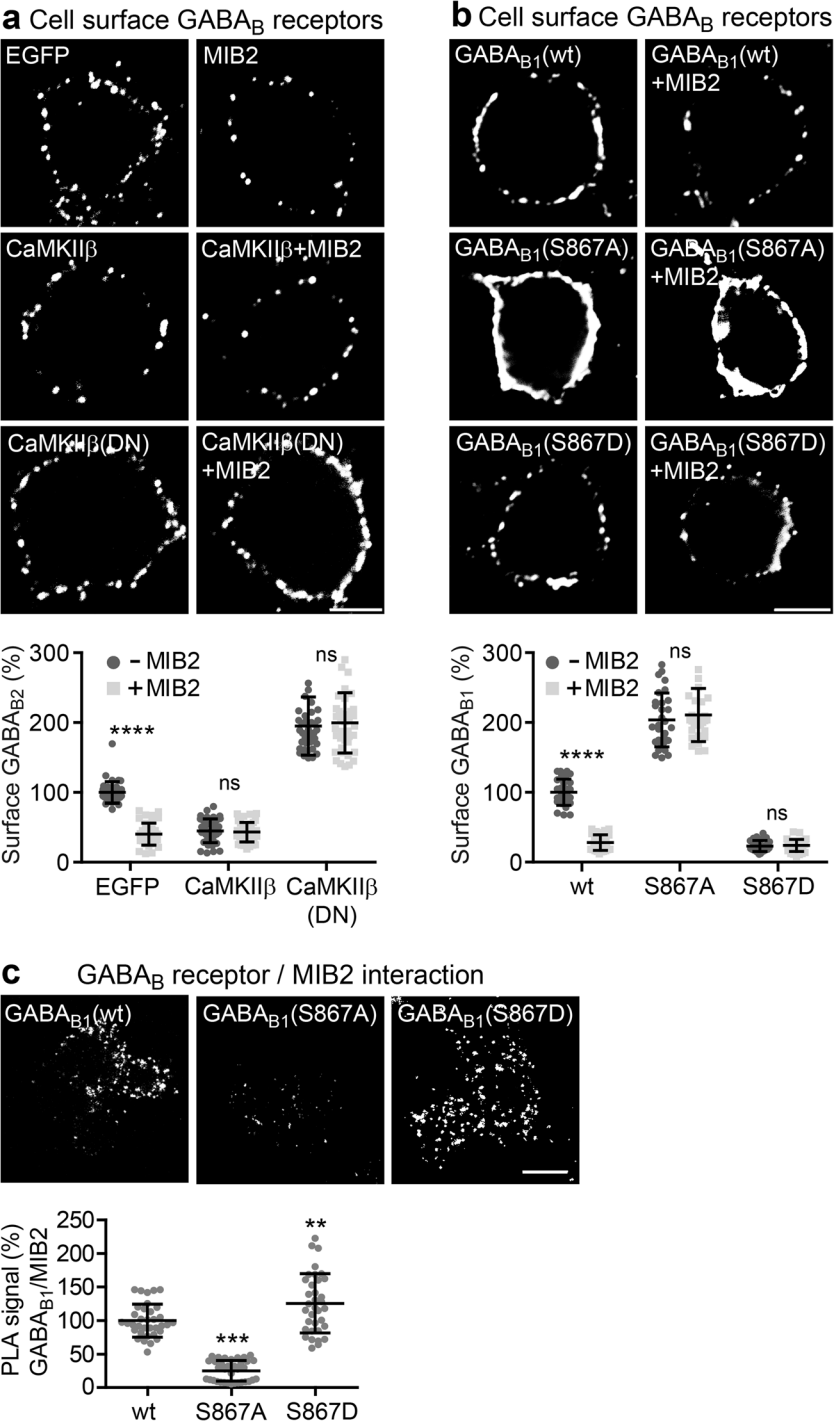
The ubiquitin E3 ligase MIB2 is involved in CaMKIIβ-mediated regulation of cell surface GABA_B_ receptors. **a** Overexpression of MIB2 in addition to CaMKII does not enhance downregulation of cell surface GABA_B_ receptors. Neurons were transfected with the indicated plasmid and tested for cell surface expression of GABA_B_ receptors using GABA_B2_ antibodies 2 days after transfection. Co-expression of MIB2 did not further enhance downregulation of cell surface receptors, indicating the involvement in the same pathway. Upper panels show representative images (scale bars, 5 μm). The graphs depict quantification of the fluorescence signals. The data represent the mean ± SD of 30 neurons per condition derived from two independent experiments. ^ns^*p* > 0.05, ****p < 0.0001; two-way ANOVA with Bonferroni’s multiple comparison test (interaction: *F*(2,234) = 32.78, *p* < 0.0001). **b** MIB2-induced downregulation of cell surface receptors depends on phosphorylation of GABA_B1_ Ser-867. Neurons were transfected with wild-type HA-tagged GABA_B1a_ (control), with the phosphorylation-deficient HA-tagged GABA_B1a_(S867A) mutant or with the phosphomimetic HA-tagged GABA_B1a_(S867D) mutant along with GABA_B2_ and with or without MIB2. Two days after transfection, neurons were tested for cell surface expression of tagged GABA_B1_ using HA antibodies. Upper panels show representative images (scale bars, 5 μm). The graph depicts quantification of the fluorescence signals. The data represent the mean ± SD of 30 neurons per condition derived from two independent experiments. ^ns^*p* > 0.05, ****p < 0.0001; two-way ANOVA, Bonferroni’s multiple comparison test (interaction: *F*(2,174) = 49.09, *p* < 0.0001). **c** GABA_B1_ phospho-mutants display altered MIB2 interaction. HEK 293 cells were transfected with wild-type GABA_B1_, GABA_B1_(S867A), or GABA_B1_(S867D) together with GABA_B2_ and MIB2. Two days after transfection, cells were analyzed for interaction of GABA_B_ receptors with MIB2 by in situ PLA using antibodies directed against GABA_B1_ and MIB2. Upper panels show representative images (scale bar, 10 μm). The graph depicts quantification of the PLA signals. The data represent the mean ± SD of 35 neurons per condition derived from two independent experiments. **p < 0.01, ***p < 0.001; one-way ANOVA, Dunnett’s multiple comparison test

We previously showed that Lys-63-linked ubiquitination of GABA_B1_ is mediated by the ubiquitin E3 ligase MIB2 [17]. We therefore tested for the involvement of MIB2 in CaMKIIβ-induced downregulation of GABA_B_ receptors. Overexpression of MIB2 or CaMKIIβ reduced cell surface expression of GABA_B_ receptors in neurons to a similar extent (MIB2: 40 ± 16%, CaMKIIβ: 45 ± 17% of EGFP-transfected control neurons; Fig. 8a). Co-transfection of MIB2 and CaMKIIβ in neurons did not further increase downregulation of the receptors (43 ± 14% of EGFP-transfected control neurons; Fig. 8a), indicating that both enzymes affect GABA_B_ receptors via the same pathway. In line with this observation, MIB2 was unable to downregulate cell surface GABA_B_ receptors when the nonfunctional CaMKIIβ mutant CaMKIIβ(DN) was simultaneously overexpressed with MIB2 (CaMKIIβ(DN): 195 **±** 42%, CaMKIIβ(DN)+MIB2: 200 **±** 43% of EGFP-transfected control neurons, Fig. 8a).

Next, we tested whether MIB2-mediated downregulation of GABA_B_ receptors depends on phosphorylation of GABA_B1_ Ser-867. Co-transfection of GABA_B1a,2_ and MIB2 in neurons strongly reduced cell surface expression of receptors containing transfected GABA_B1a_ (28 ± 11% of control, Fig. 8b). However, MIB2 failed to affect expression of the elevated or reduced cell surface expression levels of receptors containing GABA_B1a_(S867A) or GABA_B1a_(S867D) (S867A: 204 ± 39%, S867A+MIB2: 211 ± 38%, S867D: 23 ± 8%, S867D+MIB2: 24 ± 9% of control, Fig. 8b). Together, these results indicate that phosphorylation of GABA_B1_ at Ser-867 by CaMKIIβ is required for MIB2-mediated downregulation of GABA_B_ receptors.

Finally, we tested the hypothesis that CaMKII-mediated phosphorylation of GABA_B1_ might affect the interaction of MIB2 with GABA_B_ receptors. Upon transfection in HEK 293 cells, MIB2 displayed considerably lower interaction with receptors containing the phospho-deficient mutant GABA_B1a_(S867A) (25 ± 15% of control, Fig. 8c) and a higher level of interaction with receptors containing the phosphomimetic mutant GABA_B1a_(S867D) (126 ± 44% of control, Fig. 8c) than wild-type receptors. This result indicates that phosphorylation of GABA_B1_ at Ser-867 promotes the interaction of GABA_B_ receptors with MIB2.

### Sustained Activation of Glutamate Receptors Increases Lys-63-Linked Ubiquitination of GABA_B_ Receptors via Phosphorylation of GABA_B1_ Ser-867

**Fig. 9:**
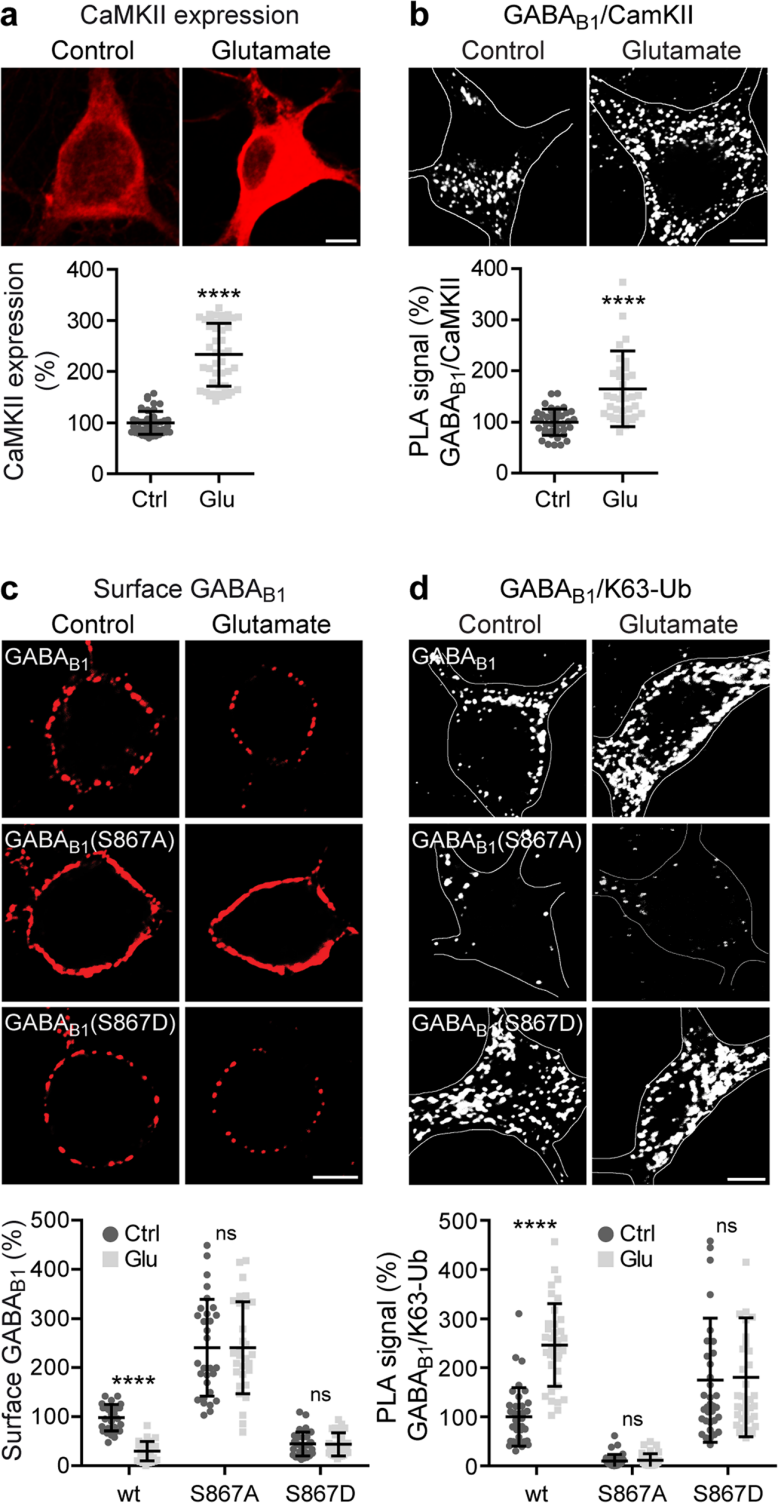
Glutamate-induced downregulation of GABA_B_ receptors. **a** Prolonged glutamate treatment increased CaMKII expression. Neurons were treated with 50 μM glutamate for 60 min and analyzed after additional 16 h for CaMKII expression. Upper panel shows representative images (scale bar, 5 μm). The data represent the mean ± SD of 45 neurons per experimental condition derived from two independent experiments. ****p < 0.0001, two-tailed unpaired *t* test. **b** Prolonged glutamate treatment increased the interaction of CaMKII with GABA_B_ receptors. Neurons were treated with 50 μM glutamate for 60 min and analyzed after additional 16 h for the interaction of CaMKII with GABA_B_ receptors via in situ *PLA* using CaMKII and GABA_B1_ antibodies. Upper panel shows representative images (scale bar, 5 μm). The data represent the mean ± SD of 39 neurons per experimental condition derived from two independent experiments. ****p < 0.0001, two-tailed unpaired *t* test. **c**, **d** Glutamate-induced downregulation of GABA_B_ receptors is mediated by GABA_B1_ Ser-867 phosphorylation-induced Lys-63-linked ubiquitination. Neurons transfected with wild-type HA-tagged GABA_B1a_, with the phosphorylation-deficient HA-tagged GABA_B1a_(S867A) mutant or with the phosphomimetic HA-tagged GABA_B1a_(S867D) mutant along with GABA_B2_ were incubated for 60 min in the absence (control) or presence of 50 μM glutamate. Neurons were analyzed for cell surface expression of transfected GABA_B1_ using HA antibodies (**c**) or analyzed for Lys-63-linked ubiquitination by in situ PLA using HA and Lys-63 antibodies (**d**). Upper panels show representative images (scale bars, 5 μm). The graphs depict quantification of the fluorescence signals (**c**) and in situ PLA signals (**d**). The data represent the mean ± SD of 28–30 (**c**) and 28–36 (**d**) neurons per condition derived from three independent experiments. ^ns^*p* > 0.05, ****p < 0.0001; two-way ANOVA, Bonferroni’s multiple comparison test (**c** interaction: *F*(2,170) = 6.27, *p* < 0.005; **d** interaction: *F*(2, 205) = 17.22, *p* < 0.0001)

Glutamate-induced downregulation of GABA_B_ receptors via lysosomal degradation depends on CaMKII-mediated phosphorylation of GABA_B1_ Ser-867 [29] as well as on Lys-63-linked ubiquitination of GABA_B1_ at Lys-697/698, Lys-892, and Lys-960 [17]. To investigate the role of CaMKII in this process, we first analyzed the effect of sustained glutamate treatment on the expression level of CaMKII as well as on the degree of CaMKII-GABA_B_ receptor interaction. Treatment of neurons for 60 min with glutamate significantly upregulated CaMKII (233 ± 61% of control, Fig. 9a) and considerably increased the interaction of CaMKII with GABA_B_ receptors (165 ± 74% of control, Fig. 9b).

We then analyzed whether glutamate-induced K63-linked ubiquitination of GABA_B_ receptors depends on phosphorylation of Ser-867. Wild-type GABA_B1_ or the two mutants GABA_B1_(S867A) and GABA_B1_(S867D) were expressed in cortical neurons and exposed for 60 min to glutamate. Under this condition, cell surface wild-type GABA_B_ receptors were downregulated to 30 ± 20% as compared to untreated controls (Fig. 9c). However, receptors containing the phospho-deficient mutant GABA_B1_(S867A) showed enhanced cell surface expression under control conditions (240 ± 99% of wild-type controls) and were not affected by glutamate (240 ± 94% of wild-type controls, Fig. 9c). In contrast, receptors containing the phosphomimetic mutant GABA_B1_(S867D) displayed strongly reduced expression under control conditions (45 ± 24% of wild-type controls, Fig. 9c) and remained unaffected after glutamate treatment (44 ± 23% of wild-type controls, Fig. 9c). A complementary pattern was observed for Lys-63-linked ubiquitination of the respective receptors as tested by in situ PLA. Treatment of cultured neurons with glutamate strongly increased K63-linked ubiquitination of wild-type GABA_B_ receptors (control: 100 ± 60%, glutamate: 247 ± 84%, Fig. 9d), whereas receptors containing the phospho-deficient mutant GABA_B1_(S867A) displayed only marginal Lys-63-linked ubiquitination under both conditions (control: 10 ± 13%, glutamate: 11 ± 13, Fig. 9d) and the phosphomimetic mutant GABA_B1_(S867D) exhibited strongly enhanced Lys-63-linked ubiquitination (control: 175 ± 126, glutamate: 181 ± 121, Fig. 9d).

These findings suggest that sustained activation of glutamate receptors induces GABA_B1_-Ser-867 phosphorylation-mediated K63-linked ubiquitination of GABA_B_ receptors, promoting their lysosomal degradation.

## Discussion

The expression of GABA_B_ receptors available for signal transduction at the cell surface critically depends on their rate of degradation. The two main cellular degradation systems—proteasomes and lysosomes—control the abundance of GABA_B_ receptors in distinct cellular compartments in response to the activity state of the neuron [10, 11, 17]. In the ER, the amount of newly synthetized GABA_B_ receptors destined for trafficking to the plasma membrane is regulated by proteasomal degradation via the ERAD machinery [10], whereas cell surface GABA_B_ receptors are degraded in lysosomes [12–16]. Both degradation pathways require ubiquitination of the receptor as targeting signals. Lys-48-linked ubiquitination of GABA_B2_ at Lys-767/771 tags GABA_B_ receptors for proteasomal degradation [10] and Lys-63-linked ubiquitination of GABA_B1_ at several sites sorts the receptors to lysosomes [17]. Interestingly, Lys-48- and Lys-63-linked ubiquitination appears to be largely segregated to GABA_B2_ and GABA_B1_ [10, 17], respectively, which might be explained by a selective interaction of the subunits with the respective E3 ligases. However, the mechanisms regulating lysosomal degradation of GABA_B_ receptors in response to changes in the physiological state of the neuron were unclear.

Under normal physiological conditions, cell surface GABA_B_ receptors constitutively internalize to early endosomes and recycle to the plasma membrane or are sorted to lysosomes for degradation [12–14, 35, 36]. Sorting the receptors to recycling or degradation must be precisely regulated in order to provide the required number of cell surface receptors for signal transduction under a given physiological condition. However, after prolonged activation of glutamate receptors, GABA_B_ receptors are rapidly downregulated by shifting the recycling/degradation balance towards lysosomal degradation [30–32]. This downregulation of GABA_B_ receptors critically depends on CaMKII-mediated phosphorylation of GABA_B1_ on Ser-867 [29] as well as on Lys-63-linked ubiquitination of GABA_B1_ mediated by the E3 ligase MIB2 [17]. As the level of CaMKII activity is regulated by the intracellular Ca^2+^ concentration, it is in an ideal position to link the increased neuronal activity after prolonged activation of glutamate receptors to lysosomal degradation of GABA_B_ receptors at least under pathological conditions. We, therefore, hypothesized that CaMKII regulates lysosomal degradation also under physiological conditions. Indeed, we found that pharmacologically blocking CaMKII significantly increased cell surface expression of GABA_B_ receptors. This increase was accompanied by a reduced co-localization of the receptors with the late endosomal marker Rab7, indicating that inhibition of CaMKII prevented sorting of GABA_B_ receptors to lysosomes. Our data also imply that a considerable fraction of GABA_B_ receptors is constitutively degraded under basal conditions since blocking CaMKII activity for only 7.5 min was sufficient to induce a significant increase (~ 150%) of cell surface GABA_B_ receptors.

CaMKII is a large protein complex constituted from 12 catalytically active subunits of different isoforms (CaMKIIα-δ) [33]. In brain, CaMKIIα and CaMKIIβ are the predominant subunits and in forebrain neurons the CaMKII holoenzyme largely consists of nine α subunits and three β subunits [37, 38]. Interestingly, we found that overexpression of CaMKIIβ, but not CaMKIIα, increased the level of GABA_B_ receptor/CaMKII interaction in an activity-dependent manner (inhibition of CaMKII activity prevented the increase in interaction), resulting in decreased cell surface receptor expression. Consistent with this observation, overexpressing its functionally inactive mutant CaMKIIβ(DN), which was expected to inhibit CaMKIIβ activity and thus lysosomal degradation of the receptors, increased cell surface expression of GABA_B_ receptors. As intracellular Ca^2+^ concentrations under basal conditions are low, this finding fits well to the observation that CaMKIIβ exhibits a ninefold higher affinity to Ca^2+^/calmodulin than CaMKIIα [38]. Thus, our data suggest that under normal physiological conditions basal CaMKIIβ activity determines the level of lysosomal degradation of GABA_B_ receptors.

Phosphorylation often regulates ubiquitination of proteins, thereby promoting their degradation [39–41]. Several lines of evidence indicate that regulation of cell surface GABA_B_ receptors by CaMKIIβ is conveyed by MIB2-mediated Lys-63-linked ubiquitination of GABA_B1_. First, overexpression in neurons of ubiquitin mutants unable to form Lys-63-linkages prevented downregulation of GABA_B_ receptors induced by overexpression of CaMKIIβ, while a mutant that only can form Lys-63-linkages, but not any other kind of linkages, did not inhibit downregulation of the receptors. Second, blocking CaMKII activity pharmacologically or by overexpression of the functionally inactive mutant CaMKIIβ(DN) reduced Lys-63-linked ubiquitination of GABA_B_ receptors. Third, the cell surface expression of three GABA_B1a_(K->R) mutants with inactivated Lys-63-linked ubiquitination sites, which are indispensable for lysosomal targeting of the receptors [17], remained unaffected by CaMKII inhibition. Fourth, overexpression of the E3 ligase MIB2, which downregulates GABA_B_ receptors via lysosomal degradation [17], neither enhanced CaMKIIβ-mediated downregulation of cell surface receptors nor affected the expression of a GABA_B1_ mutant with inactivated CaMKII phosphorylation site (GABA_B1_(S867A)). Finally, our experiments with the CaMKII phosphorylation-deficient and phosphomimetic GABA_B1_(S867) mutants indicate that direct phosphorylation of GABA_B1_ Ser-867 promotes Lys-63-linked ubiquitination and thereby degradation of GABA_B_ receptors. Under normal physiological as well as conditions of over-excitation via prolonged activation of glutamate receptors, the phospho-deficient mutant GABA_B_(S867A) displayed only marginal Lys-63-linked ubiquitination and increased cell surface expression whereas the phosphomimetic mutant GABA_B1_(S867D) exhibited strongly increased Lys-63-linked ubiquitination and reduced cell surface expression.

The precise mechanism by which CaMKIIβ promotes Lys63-linked ubiquitination of GABA_B1_ remains unresolved. Phosphorylation of GABA_B1_(S867) might be the signal that targets the receptors to an endosomal compartment where they are Lys-63-linked ubiquitinated by MIB2. Alternatively, phosphorylation of GABA_B1_(S867) might induce a conformational change in the receptor that uncovers the MIB2 interaction site or exposes the lysine residues for ubiquitination. We favor the latter hypothesis, which is supported by our finding that MIB2 displayed increased interaction with the phosphomimetic mutant GABA_B1_(S867D) and reduced interaction with the phospho-deficient mutant GABA_B1_(S867A). However, further experimentation is required to reveal the mechanism as to how CaMKIIβ promotes Lys-63-linked ubiquitination of GABA_B1_.

In addition to CaMKIIβ controlling cell surface GABA_B_ receptor expression under normal physiological conditions, it rapidly downregulates the receptors upon sustained activation of glutamate receptors [29–32], a pathological condition that occurs in cerebral ischemia and leads to excitotoxic cell death [42, 43]. Downregulation of GABA_B_ receptors is triggered by increased Ca^2+^ influx through NMDA receptors and voltage-gated Ca^2+^ channels [30], leading to enhanced CaMKII-mediated phosphorylation of GABA_B1_ on Ser867 [29]. This causes the re-routing of GABA_B_ receptors from the normal constitutive recycling pathway to lysosomal degradation. Our present results show that this pathological downregulation of GABA_B_ receptors is maintained after removal of glutamate due to the upregulation of CaMKII expression and the increased interaction of CaMKII with GABA_B_ receptors. Therefore, interfering with this pathway to normalize GABA_B_ receptor-mediated inhibition might be a potential approach to counteract over-excitation and to limit neuronal death.

In conclusion, our data indicate that phosphorylation of GABA_B1_ at Ser-867 by CaMKIIβ induces MIB2-mediated K63-linked ubiquitination of GABA_B1_ at multiple sites, which sorts GABA_B_ receptors to lysosomes for degradation. This mechanism is expected to fine-tune cell surface expression of GABA_B_ receptors under physiological conditions and to considerably affect receptor expression in diseases associated with disturbed Ca^2+^ homeostasis.
